# Multiple Phosphatases Regulate Carbon Source-Dependent Germination and Primary Metabolism in *Aspergillus nidulans*

**DOI:** 10.1534/g3.115.016667

**Published:** 2015-03-11

**Authors:** Leandro José de Assis, Laure Nicolas Annick Ries, Marcela Savoldi, Taisa Magnani Dinamarco, Gustavo Henrique Goldman, Neil Andrew Brown

**Affiliations:** * Faculdade de Ciências Farmacêuticas de Ribeirão Preto, Universidade de São Paulo, Brazil CEP14040-903; † Faculdade de Filosofia Ciências e Letras de Ribeirão Preto, Universidade de São Paulo, Brazil CEP14040-901; ‡ Laboratório Nacional de Ciência e Tecnologia do Bioetanol, CTBE-CNPEM, Brazil CEP13083-970

**Keywords:** phosphatase, germination, glucose metabolism, cell cycle

## Abstract

*Aspergillus nidulans* is an important mold and a model system for the study of fungal cell biology. In addition, invasive *A. nidulans* pulmonary infections are common in humans with chronic granulomatous disease. The morphological and biochemical transition from dormant conidia into active, growing, filamentous hyphae requires the coordination of numerous biosynthetic, developmental, and metabolic processes. The present study exhibited the diversity of roles performed by seven phosphatases in regulating cell cycle, development, and metabolism in response to glucose and alternative carbon sources. The identified phosphatases highlighted the importance of several signaling pathways regulating filamentous growth, the action of the pyruvate dehydrogenase complex as a metabolic switch controlling carbon usage, and the identification of the key function performed by the α-ketoglutarate dehydrogenase during germination. These novel insights into the fundamental roles of numerous phosphatases in germination and carbon sensing have provided new avenues of research into the identification of inhibitors of fungal germination, with implications for the food, feed, and pharmaceutical industries.

*Aspergillus nidulans* is an important mold and a model system for the study of fungal cell biology. In addition, invasive *A. nidulans* pulmonary infections are common in humans with chronic granulomatous disease, resulting in a high rate of mortality (Henriet *et al.* 2012). Reproduction in *A. nidulans* yields asexual conidia and sexual ascospores that are essential for fungal dispersal and the survival of harsh environments. Spore dispersal and germination is a key factor in the spoilage of food and pathogenicity (Klich 2009). Dormant conidia can remain viable for years until the appropriate conditions for germination are detected, including the exogenous presence of water, salt, carbon, and nitrogen sources (Osherov and May 2000, 2001). Conidial germination is characterized by two morphological steps, isotropic growth (conidial swelling) and the establishment of polarization and germ-tube emergence (D’Enfert 1997).

Dormant *A. nidulans* conidia are arrested in the G1 of the cell cycle (Bergen and Morris 1983). With the detection of conditions suitable for germination, the cell cycle is reinitiated. Entry into the S phase occurs during isotropic growth and the first round of mitosis occurs before polarization and germ-tube emergence (Bergen and Morris 1983; Momany and Taylor 2000). Hence, germination involves the detection of external nutrients, which results in primary metabolism shifting toward energy-yielding reactions that sequentially use intracellular stores prior to extracellular sources, as well as the initiation of the cell cycle and the biosynthesis of cellular components, and the differentiation of growth morphology. The signaling networks regulating the aforementioned processes involved in germination are unclear. An enhanced understanding of these signaling pathways would have a wide ranging impact on fungal biology and subsequently represents the focus of this study.

*A. nidulans* can germinate and sustain growth on a diverse range of simple and complex carbon sources, including saccharides, alcohols, proteins, and lipids. This metabolic flexibility has endowed it with the ability to adjust metabolism and nutrient uptake to fit the encountered environment (MacCabe 2003). Glucose metabolism represents the greatest energetic gain and is the preferred carbon source for the majority of microbes. The ability to sense intracellular or extracellular nutrient sources enables the coordination of cellular metabolism and the preferential consumption of glucose prior to alternative carbon sources, referred to as carbon catabolite repression (CCR) (Brown *et al.* 2014). Primary glucose metabolism is well-characterized in fungi where the carbon locked within the glucose is directed toward energy-producing reactions such as its oxidation via glycolysis and the tricarboxylic acid (TCA) cycle, fermentation, and the production of ethanol, or alternatively the production of storage sugars such as trehalose and glycerol (Figure 1). During the isotropic growth phase of germination, internal trehalose and mannitol are metabolized (D’Enfert 1997; D’Enfert *et al.* 1999; Fillinger *et al.* 2002) prior to the uptake and metabolism of the external carbon source, which triggered the breaking of dormancy (MacCabe 2003). Therefore, during the short period of conidial germination, primary metabolism is shifted multiple times.

**Figure 1:**
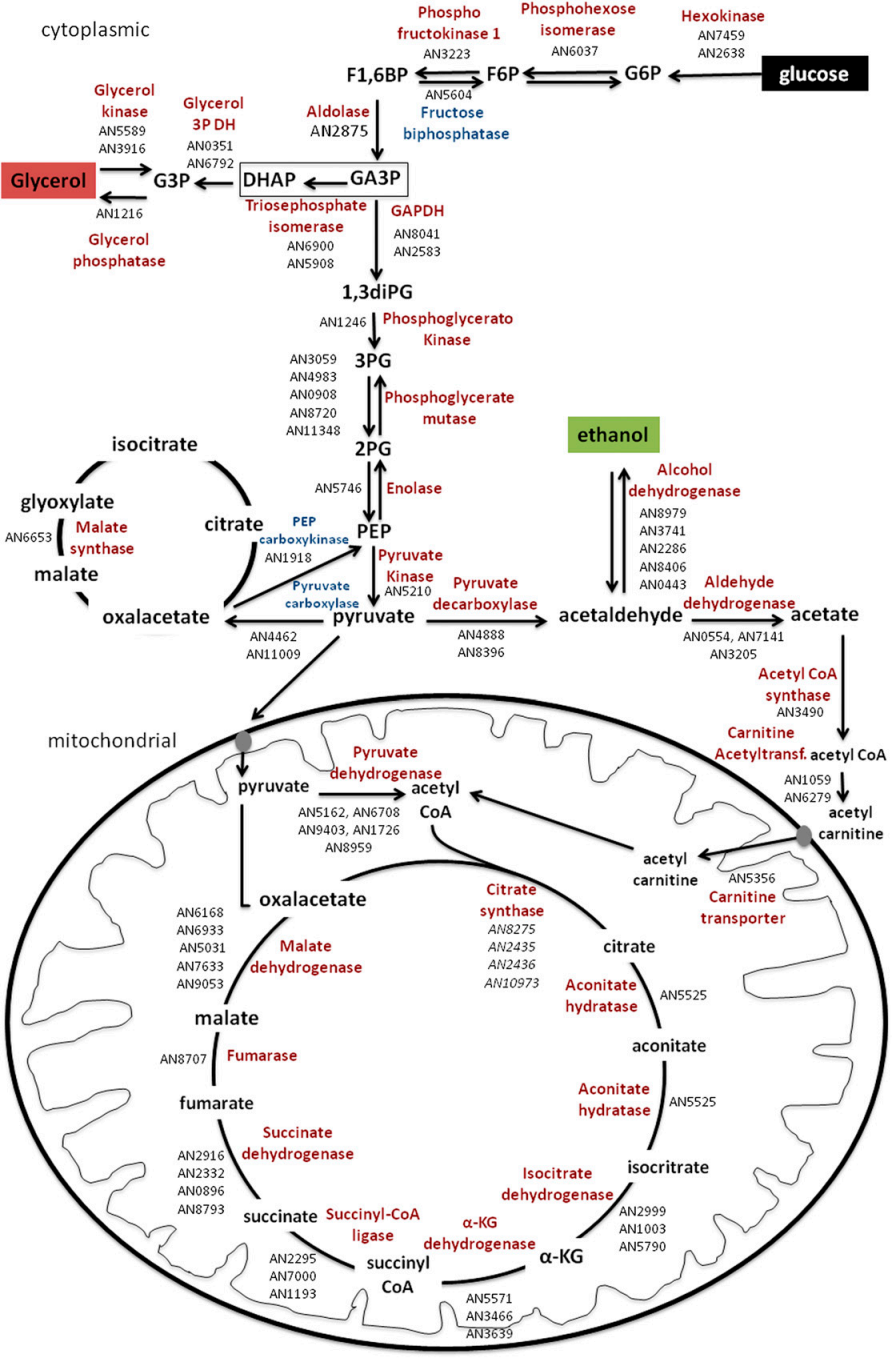
Primary carbon metabolism in *A. nidulans* and the representative genes involved in each biochemical step of glycolysis, glycerol metabolism, fermentation, TCA cycle (represented in red), and gluconeogenesis (represented in blue). G6P = glucose 6 phosphate; F6P = fructose 6 phosphate; F1,6BP = fructose 1,6 biphosphate; GA3P = glyceraldehyde 3 phosphate; DHAP = dihydroxyacetone phosphate; G3P = glycerol 3 phosphate; 1,3diPG = 1,3 diphosphoglycerate; 3PG = 3 phosphoglycerate; 2PG = 2 phosphoglycerate; PEP = phosphoenolpyruvate; DH = dehydrogenase.

Nutrient sensing and the downstream signaling cascades have a profound impact on the regulation of biochemical metabolic pathways, biosynthetic processes, and developmental changes. Signaling cascade intermediates are subject to post-translational modifications that influence target protein activity, localization, and function, such as protein phosphorylation, mediated by the opposing function of protein kinases and phosphatases (Shi 2009). In *Saccharomyces cerevisiae*, the cAMP-dependent protein kinase A (PKA) pathway mediates glucose sensing, promoting glucose uptake and utilization while inhibiting the sucrose nonfermenting kinase (Snf1p)-mediated alternative carbon usage pathway and influencing cell cycle progression (Farkas *et al.* 1989; Dong *et al.* 1995; Pedruzzi *et al.* 2003; Barrett *et al.* 2012).

In filamentous fungi, such as *A. nidulans*, the G-protein-coupled receptor-mediated and Ras-mediated cAMP-PKA pathways regulate germination and trehalose metabolism, whereas the SchA kinase performs parallel functions (Fillinger *et al.* 2002; Lafon *et al.* 2005, 2006). Despite advancements in the understanding of the *A. nidulans* phospho-proteome and the roles played by several central protein kinases, such as PkaA, SchA, and SnfA (Fillinger *et al.* 2002; Brown *et al.* 2013; Ramsubramaniam *et al.* 2014), current knowledge of the functions performed by the opposing phosphatases in carbon metabolism and germination is scarce. The recent generation of an *A. nidulans* null mutant collection containing 103 nonessential protein kinases (NPKs) and 28 nonessential protein phosphatases (NPPs) has assisted in the dissection of the signaling pathways involved in fungal development and cell cycle (Son and Osmani 2009; De Souza *et al.* 2013). A subsequent screen of the *A. nidulans* NPK and NPP null mutant collection identified NPKs and NPPs involved in the regulation of CCR and cellulose utilization (Brown *et al.* 2014). Additionally, seven NPPs were shown to be unable to utilize glucose as a sole carbon source. An enhanced understanding of how these seven NPPs influenced carbon sensing, metabolism, and germination in *A. nidulans* would be of great interest. Hence, the present study of *A. nidulans* characterized the different functions of these seven NPPs in carbon sensing. This investigation showed the essentiality of multiple NPPs in the regulation of shared and distinct processes that impacted on primary carbon metabolism, cell cycle, and, ultimately, germination.

## Materials and Methods

### Strains and culture conditions

The two *A. nidulans* strains FGSC A4 (*pyrG89*; *wA3*; *pyroA4*; *veA1*) and TN02A3 (*pyrG89*; *pyroA4*; *nkuA*::*argB*) were used as reference for comparisons with the NPP mutant collection. The 28 NPP null mutants were created by *in vitro* recombination in *S. cerevisiae* followed by *A. nidulans* protoplast transformation (http://www.fgsc.net/Aspergillus/KO_Cassettes.htm) (Son and Osmani 2009; De Souza *et al.* 2013). All fungal strains were propagated on complete media (2% w/v glucose, 0.5% w/v yeast extract, trace elements) or minimal media (1% w/v glucose or hydrolyzed casein (CA), trace elements, supplemented with pyridoxine, riboflavin, and uracil/ uridine, pH 6.5) plus or minus 2% w/v agar.

### Screening for growth defects and sensitivity to 2-DG

For all radial growth experiments, the respective strains were point-inoculated onto minimal media agar plates (±1% glucose) supplemented with 1% alternative carbon source. For pre-incubation experiments, 1×10^7^ conidia were incubated in liquid minimal media supplemented with 1% CA for 4 hr prior to inoculating a 5-μl droplet, or point of inoculation, onto solid minimal media plus 1% glucose. Plates were incubated for 48 hr at 37° before examination. The toxic glucose analog, 2-deoxyglucose (2-DG; Sigma), was used to measure sensitivity to CCR via growing the strains in minimal media plus CA (1%) and increasing concentrations of 2-DG (25–100 µM) for 96 hr at 37°. In addition, to observe the post-germination influence of 2-DG, conidia were pre-incubated in CA for 4 hr and then inoculated onto 1% CA plus 2-DG (5-60 µM) for 48 hr at 37°.

To map the block in carbon metabolism, all strains were inoculated onto media containing combinations of amino acids (50 mM each amino acid) that enter into the tricarboxylic acid (TCA) cycle at specific steps: pyruvate (alanine and glycine), acetyl-CoA (leucine and lysine), ketoglutarate (arginine, proline, histidine and glutamine), succinyl-CoA (isoleucine, valine, methionine, and threonine), fumarate (tyrosine and phenylalanine), and oxaloacetate (asparagine and aspartate) as a sole carbon source for 96 hr at 37°.

### Double reciprocal shift assays

The reciprocal shift experiments used to determine the phase of the cell cycle at which the phosphatase mutants arrested were performed as described previously (Bergen *et al.* 1984). Briefly, conidia from the phosphatase mutants were inoculated on cover slips and incubated in minimal media plus 1% glucose for 7 hr at 37°. Untreated germlings were fixed (as a control), transferred to minimal media plus 1% glucose for 3 hr, or transferred to minimal media plus 1% CA with 25 mM hydroxyurea (HU) for 3 hr. For the reciprocal experiments, conidia were arrested in S-phase by incubation in minimal media plus 1% CA containing 25 mM HU for 7 hr at 37°. The cover slips were either fixed (as a control) or transferred to minimal media plus 1% CA without HU or shifted to minimal media plus 1% glucose without HU. Cover slips were stained with Hoechst (3 µg/ml) for 2 min and washed in phosphate-buffered saline (PBS; 1×, NaCl 8 g/liter, KCl, 0.2 g/liter, Na_2_HPO_4,_ 1.44 g/liter, and KH_2_PO_4_ 0.24 g/liter, pH 7.4). Subsequently, the number of nuclei per germling was counted for 80 to 120 germlings.

### Glucose uptake

Minimal media cultures (30 ml) containing 1% CA as a carbon source were inoculated with 3×10^6^ conidia and subsequently incubated for 24 h rat 37° in a rotary shaker (180 rpm). The mycelia were then transferred to minimal medium plus 1% glucose as a carbon source for an additional 24 hr. The amount of free glucose remaining in the medium was measured using the Glucose GOD-PAP Liquid Stable Mono-reagent kit (LaborLab Laboratories Ltd. Guarulhos, São Paulo, Brazil), according to the manufacturer’s instructions. Glucose uptake was calculated via determining the difference in glucose present in the initial media and after 24-hr incubation.

### Oxygen uptake measurements

Minimal media cultures (30 ml) containing 1% CA as a carbon source were inoculated with 3×10^6^ conidia and subsequently incubated for 24 hr at 37° in a rotary shaker (180 rpm). The mycelia were then transferred to minimal medium plus 1% glucose as a carbon source for an additional 24 hr. The mycelia were harvested by centrifugation, and the oxygen consumption was measured by a Clark-type electrode (Hansatech Instruments Ltd Oxygen Electrode Measurement Systems) in 1 ml of minimal medium supplemented with 1% glucose. Measurements were normalized using mycelial dry weight (Dinamarco *et al.* 2010).

### RNA extraction and RT-qPCR

Mycelia were harvested by vacuum filtration and immediately frozen in liquid nitrogen. Mycelia were ground into a fine powder in liquid nitrogen and the total RNA was extracted using TRIZOL (Invitrogen) before being treated with RNAse-free DNAse (Promega) and purified with the RNeasy Mini Kit (Qiagen). RNA integrity was confirmed using the Bioanalyser Nano Kit (Agilent Technologies) and the Agilent Bioanalyser 2100. The SuperScript III First Strand Synthesis system (Invitrogen) and oligo(dT) primers were used for cDNA synthesis according to the manufacturer’s instructions. All RT-qPCR reactions were performed using an ABI 7500 Fast Real-Time PCR System and Taq-Man Universal PCR Master Mix kit (Applied Biosystems). The tubulin gene *tubC* was used as an endogenous control. The primers used for the glucose transporter and metabolic enzyme encoding genes are listed in Supporting Information, Table S1.

### Pyruvate, ethanol, glycerol, and trehalose measurements in mycelia

Mycelia of the respective strains were grown under the same experimental conditions as described for the glucose uptake investigation. Mycelia were ground in liquid nitrogen and immediately resuspended by inversion in extraction buffer [50 mM: Tris base pH 7.0, 50 mM NaF, 1 mM Na_3_VO_4_, 1 mM DTT, phosphatase inhibitor cocktail P0044 (Sigma), and an EDTA-free protease inhibitor cocktail (Roche)] prior to centrifugation for 5 min at 14,000*g*. The protein concentration of the extracts was measured using the Bio-Rad protein assay according to manufacturer’s instructions. The glycerol, pyruvate, and trehalose content within the extracted cell lysate (equivalent to 5, 10, and 20 μg of total protein for the respective assays) was measured using the Free Glycerol Detection ab65337 kit (AbCam) according to the manufacturer’s instructions. The pyruvate was measured by performing a coupled reaction using lactate dehydrogenase (LDH) and nicotine adenine dinucleotide in its reduced form (NADH) (Chretien *et al.* 1995). This reaction converted pyruvate to lactate by the oxidation of NADH, resulting in a measurable decline in absorbance at 340 nm. The trehalose content was measured using Trehalose Assay kit K-TREH 11/12 (Megazyme) according to the manufacturer’s instructions with an additional standard curve ranging from 0 to 4 μg of trehalose dihydrate. Ethanol concentration was determined by measuring the increased concentration of NADH at 340 nm in the coupled reaction using alcohol dehydrogenase, pyrophosphate, glycine, semicarbazide, and nicotine adenine dinucleotide (NAD^+^) as described previously (de Assis *et al.* 2013). Absorbance at 340 nm was detected using a SpectraMax I3 spectrometer (Molecular Devices).

### Trehalose measurements in germinating conidia

Trehalose was extracted from 5×10^7^ fresh conidia, plus conidia germinating in minimal media containing either glucose or CA as a single carbon source. Conidia were incubated for 150 min at 37° in 1 ml of the respective media, collected by centrifuge for 5 min at 14,000*g* at 4°, and washed once using 1 ml of water. The conidia were broken using 0.3 g of glass beads (0.65 μm) and 1 ml of buffer extraction [50 mM Tris base pH 7.0, 50 mM NaF, 1 mM Na_3_VO_4_, 1 mM DTT, phosphatase inhibitor cocktail P0044 (Sigma) and an EDTA-free protease inhibitor cocktail (Roche)]. The trehalose content was measured using Trehalose Assay kit K-TREH 11/12 (Megazyme) according to the manufacturer’s instructions with additional standard curve (0–4 μg) of the trehalose dihydrate.

### Alpha-ketoglutarate assay

Total cell lysate was prepared as described in the previous section. Alpha-ketoglutarate activity was measured in 30 μg of the cell lysates (Chretien *et al.* 1995; Tretter and Adam-Vizi 2004; Habelhah *et al.* 2004), with modifications to the reaction buffer (100 mM Tris-HCl pH 7.0, 5 mM 2-mercapto ethanol, 1 mM MgCl_2_, 2 mM TPP, 5 mM alpha-ketoglutarate acid disodium salt, 1 mM NAD^+^ and 0.2 mM Coenzyme A). The generation of the reduced form of NADH was measured at 340 nm using a SpectraMax I3 spectrometer (Molecular Devices).

### ADP/ATP ratio measurement

Mycelia of the respective strains were grown under the same experimental conditions as described for the glucose uptake investigation. Total cell lysate was prepared as described in the previous section and the ADP/ATP ratio was measured in 10 μg of the cell lysate using the ADP/ATP ratio assay kit MAK135 (Sigma) following the manufacturer’s instructions and was read using a SpectraMax I3 spectrometer (Molecular Devices).

### Isotropic growth measurements

Fresh conidia were harvested in ultapure sterile water and immediately observed by microscopy, and the radial diameter was measured immediately (control) or after incubation for 2 hr in minimal media plus glucose as a carbon source (treatment). The radial diameter was used to calculate the volume of conidia before and after treatment, following the assumption that the conidia were spherical, with the following formula (V = 4/3 .π.R^3^).

### Statistical analysis of biochemical assays

Statistical analyses were performed on three biological replicates using a one-tailed *t*-test (Prism, GraphPad) with significance levels of *P < 0 0.05, **P < 0.01, and ***P < 0.001.

### Microarray analysis

Initially 1×10^7^ conidia were incubated in minimal media containing 1% CA as a carbon source at 37° in a rotatory shaker (180 rpm) for 24 hr. Subsequently, mycelia were washed with sterile water and then incubated in minimal media plus 1% glucose at 37° for an additional 4 hr. At each step the mycelia from three biological replicates were collected by vacuum filtration and immediately frozen in liquid nitrogen. Agilent custom-designed oligonucleotides arrays (Colabardini *et al.* 2014) were used to identify the transcriptional differences during growth on CA (Cy3 reference) and glucose (Cy5) for the wild-type and *ΔptpB* strains. Total RNA was extracted and RNA integrity was confirmed as described previously. Array hybridization and data analysis were performed according to Colabardini *et al.* (2014). The dataset was deposited in the Gene Expression Omnibus (http://www.ncbi.nlm.nih.gov/geo/query/acc.cgi?acc=GSE61980). Genes were determined as differentially expressed between carbon sources by applying a *t*-test (*P* < 0.01) performed within the Mev software (Saeed *et al.* 2003). The differentially expressed genes were divided into those that were upregulated or downregulated and strain-specific transcriptional alterations were identified via Venn analysis. The functional profile and identification of overrepresented GO terms within the differentially expressed gene sets from each strain under the two nutritional conditions were identified using the FetGOat software (http://www.broadinstitute.org/fetgoat/index.html) and FunCat (http://pedant.gsf.de/pedant3htmlview/pedant3view?Method=analysis&Db=p3_p130_Asp_nidul).

## Results

### Identification of seven NPPs that play important roles in carbon source–dependent germination

To identify NPPs involved in glucose sensing and CCR, a collection of 28 *A. nidulans* NPP null mutants was previously screened for an altered ability to grow on either glucose or cellulose as a sole carbon source. Seven NPP null mutants were shown to be unable to grow directly on glucose (Brown *et al.* 2013). BLASTp analyses of the seven NPP encoding genes identified the phosphatase subfamily and the orthologous genes in *S. cerevisiae* (Table 1) that have been shown to regulate the mitochondrial pyruvate dehydrogenase complex (paralogs *PTC5* and *PTC6*), the pheromone and cell wall integrity MAPK pathways (paralogs *MSG5* and *SDP1*), the Msn2p-mediated general stress response (*PSR1*), filamentation (*PTP1*), and cell cycle (*PPS1*). The *A. nidulans* phosphatases were named accordingly (Table 1).

**Table 1:** Seven nonessential *A. nidulans* phosphatases null mutants

*A. nidulans* Genes	Family*^a^*	Subfamily*^b^*	Class/Domain*^c^*	*S. Cerevisiae* Gene	E Value	Identity (%)
AN4544 (*msgA*)	PTP	Dual-specificity	DSP	*MSG5*	1e-22	30
AN0129 (*ppsA*)	PTP	Dual-specificity	DSP	*PPS1*	1e-51	31
AN10077 (*psrA*)	S/T	Asp-based	HAD	*PSR1*	3e-92	69
AN0914 (*ptcD*)	S/T	PPM	PP2C	*PTC6*	1e-25	33
AN5722 (*ptcE*)	S/T	PPM	PP2C	*PTC5*	5e-112	44
AN4896 (*ptpB*)	PTP	Classical	PTP	*PTP1*	3e-39	33
AN10138 (*sdpA*)	PTP	Dual-specificity	DSP	*SDP1*	9e-05	33

Seven nonessential *A. nidulans* phosphatases null mutants that cannot germinate on glucose as a sole carbon source and the homologous functionally characterized *S. cerevisiae* phosphatases.

a Family abbreviations: S/T, serine/threonine; PTP, protein tyrosine phosphatase.

b Subfamily abbreviations: Asp-based, aspartate-based phosphatase.

c Class/domain abbreviations: PP2Cc, protein phosphatase 2C catalytic subunit; PPM, Mg2^+^ or Mn2^+^-dependent protein phosphatase; HAD, haloacid dehalogenase; PTP, protein tyrosine phosphatase catalytic subunit; DSPc, dual-specificity phosphatase catalytic subunit.

**Figure 2:**
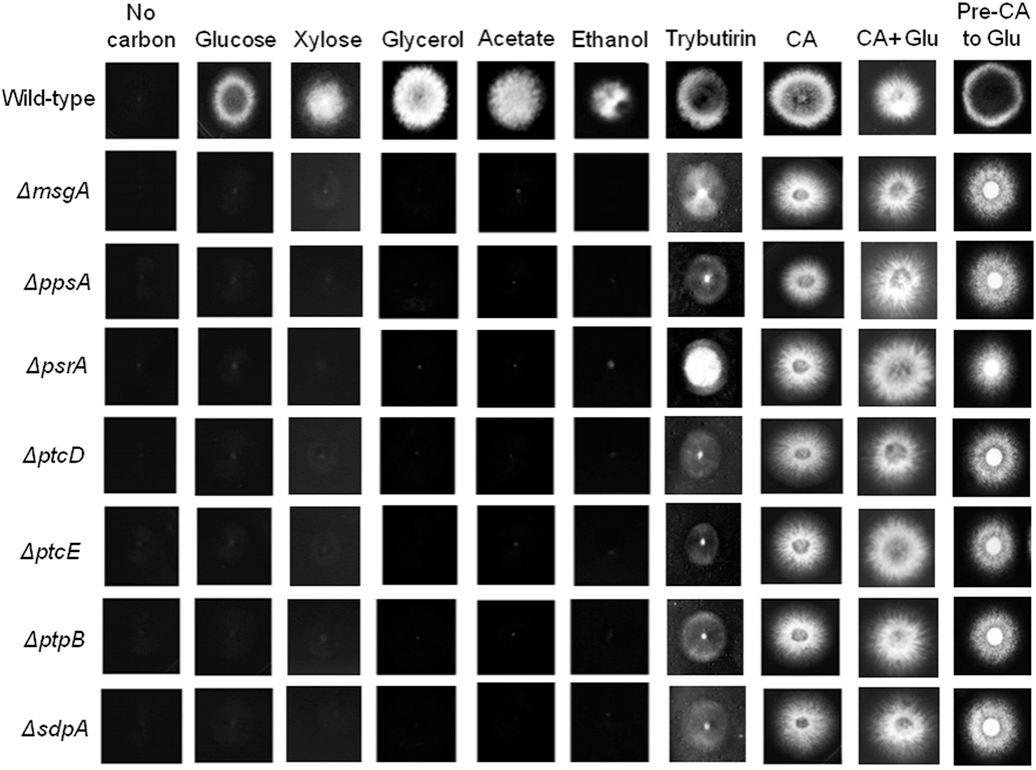
The growth of the seven NPP null mutants and the wild-type strain (FGSC A4) in the presence of various carbon sources. Fungal strains were grown directly on minimal media agar plates supplemented with 1% of the respective carbon source, except for the final column where conidia were pre-incubated in CA for 4 hr prior to plating on glucose. All cultures were incubated at 37° for 48 hr. CA, hydrolyzed casein; CA+Glu, hydrolyzed casein+glucose; and Pre-CA to Gluc, pre-grown in hydrolyzed casein and transferred to glucose.

The seven NPP mutants were also unable to grow directly on xylose, glycerol, ethanol, and acetate as sole carbon sources, but they were able to grow directly on hydrolyzed casein (CA) or tributyrin and media containing both CA and glucose (Figure 2). This suggested that the seven NPP mutants possessed defects in the sensing and/or metabolism of carbon sources that entered primary carbon metabolism prior to the TCA cycle but were able to grow on amino acids and lipids that entered primary carbon metabolism as TCA cycle intermediates.

To validate the involvement of the identified NPPs in glucose signaling, metabolism, and CCR during germination, the seven NPPs were grown directly on CA plus an increasing concentration of 2-DG, a toxic glucose analog that mimics glucose signaling but cannot be metabolized (Figure S1). Compared to the wild-type strain, *ΔpsrA* showed similar 2-DG sensitivity, *ΔppsA* was moderately more resistant to 2-DG, whereas *ΔmsgA*, *ΔptcD*, and *ΔptcE* were significantly more resistant to 2-DG. This suggests that the latter three strains may possess impaired glucose sensing or glucose uptake pathways. Conversely, *ΔptpB* and *ΔsdpA* exhibited a significant and moderate increase in sensitivity to 2-DG, respectively. This implied that these two NPP mutants were unable to grow on glucose due to a deficiency in glucose metabolism and not a specific issue in glucose sensing or uptake.

A similar approach was used to evaluate the post-germination defects in glucose sensing and/or metabolism, where the wild-type and NPP mutants were pre-incubated in CA for 4 hr and transferred to CA plus increasing concentrations of 2-DG. All the NPP mutants showed increased resistance to 2-DG compared to the wild-type strain, especially *ΔsdpA* (Figure S2). This demonstrated that all strains possessed defects in glucose sensing and/or metabolism after germination.

In *A. nidulans*, conidial germination requires the sensing of the appropriate conditions to support growth resulting in the breaking of dormancy, as represented by isotropic growth, whereas the subsequent polarization and germ-tube emergence is an energy-consuming process (Fillinger *et al.* 2002; MacCabe 2003; Hayer *et al.* 2014). To assess if the NPP mutants were unable to grow on glucose due to a defect in germination, conidia were pre-incubated in liquid media containing CA as a carbon source for 4 hr prior to plating on solid media containing glucose as a sole carbon source. Pre-incubation in CA restored the ability of all seven NPP mutants to grow on glucose (Figure 2). However, the seven NPP mutants exhibited reduced growth on glucose after germination on CA compared to the wild-type strain (Table 2). This suggested that these NPP mutants possessed defects in carbon sensing and/or metabolism, mechanisms that were essential for germination, in addition to post-germination defects in carbon metabolism.

**Table 2:** Growth after germination (dry weight) of the seven NPP mutants and the wild-type strain on glucose

	Fungal Strain							
Trait	WT	*ΔmsgA* AN4544	*ΔppsA* AN0129	*ΔpsrA* AN10077	*ΔptcD* AN0914	*ΔptcE* AN5722	*ΔptpB* AN4896	*ΔsdpA* AN10138
Pre-CA for 4 hr and transfer to glucose 24 hr (dry weight in grams)	0.056 ± 0.002	0.033** ± 0.004	0.032** ± 0.001	0.033** ± 0.005	0.033*** ± 0.002	0.033** ± 0.001	0.030*** ± 0.003	0.035*** ± 0.002

1×107 conidia pre-germinated in CA minimal media for 4 hr prior to transfer to glucose minimal media for 24 hr. Level of significance compared to the wild-type strain: *P < 0.05, **P < 0.01, and ***P < 0.001.

### NPPs influence glucose-dependent breaking of conidial dormancy and reinitiation of cell cycle

To further evaluate the impact of the seven NPP mutants on germination, the ability to break dormancy and produce isotropic growth in glucose-containing media was assessed (Table 3). The *ΔptcE* mutant was unable to induce swelling, suggesting a major defect in the detection of glucose, whereas the *ΔptcD*, *ΔpsrA*, and *ΔsdpA* mutants also showed a significant reduction in conidial swelling. The *ΔmsgA*, *ΔptpB*, and *ΔppsA* mutants showed a significant increase in conidial swelling (>75%) and were therefore unlikely to possess major defects in glucose detection.

**Table 3:** Quantification of isotropic growth during germination of the *A. nidulans* phosphatase null mutants on glucose as a sole carbon source

Fungal Strain	Conidial Diameter (μm)		Conidial Volume (μm^3^)		Swelling (%)	t test*^a^*
	Fresh (0 hr)	Glucose (2 hr)	Fresh (0 hr)	Glucose (2 hr)		
WT	3.7 ± 0.04	5.4 ± 0.06	26.8 ± 7.16	81.1 ± 20.59	202.4 ± 15.23	—
*ΔmsgA*	3.7 ± 0.03	4.5 ± 0.05	26.7 ± 6.50	46.7 ± 15.63	75.1 ± 18.99	0.0001
*ΔppsA*	4.1 ± 0.06	5.2 ± 0.08	36.6 ± 16.86	75.0 ± 29.94	105.0 ± 15.23	0.01
*ΔpsrA*	3.8 ± 0.02	4.1 ± 0.04	27.8 ± 5.88	35.0 ± 9.44	25.5 ± 4.70	0.0001
*ΔptcD*	3.8 ± 0.03	4.0 ± 0.05	27.5 ± 6.18	34.1 ± 10.66	24.2 ± 6.03	0.0001
*ΔptcE*	3.8 ± 0.03	3.5 ± 0.05	27.9 ± 7.30	23.1 ± 8.70	0	0.0001
*ΔptpB*	3.7 ± 0.03	4.7 ± 0.04	26.9 ± 7.90	55.1 ± 14.78	105.0 ± 6.86	0.0001
*ΔsdpA*	3.6 ± 0.03	4.2 ± 0.05	25.7 ± 6.68	37.4 ± 14.80	45.6 ± 8.68	0.0001

Presented are the conidial diameter and volume before and after incubation in glucose media for 2 hr plus the percentage change in conidial volume.

a t, test wild-type *vs.* null mutant strain for swelling.

Reciprocal shift assays were performed to determine at which stage of interphase the *ΔmsgA*, *ΔptpB*, and *ΔppsA* mutants were arrested (Table 4). Wild-type *A. nidulans* dormant conidia predominately arrest in or prior to S-phase, whereas a small subpopulation, approximately 10%, contains more DNA and may be binuclear (Bergen and Morris 1983). When conidia from the three NPP mutants were incubated in glucose, only 4–8% of nuclei underwent mitotic division. However, after transfer to CA, 54% and 63% of the *ΔptpB* and *ΔppsA* germlings had multiple nuclei, demonstrating the completion of at least a single turn of the cell cycle. Shifting the *ΔptpB* and *ΔppsA* conidia from glucose to CA plus hydroxyurea (HU), which blocks cell cycle progression in S-phase, revealed that *ΔptpB* was unable to perform mitosis, with nearly all conidia still possessing a single nucleus, whereas *ΔppsA* was able to complete the cell cycle with the majority of germlings (70%) containing multiple nuclei. Alternatively, transfer of the *ΔmsgA* conidia from glucose to CA resulted in a delayed reactivation of the cell cycle, whereas prolonged incubation in CA plus HU did not permit cell cycle progression and an increase in nuclei number.

**Table 4:** Double reciprocal shift assay for the *ΔptpB*, *ΔmsgA*, and *ΔppsA A. nidulans* phosphatase mutants

Fungal Strain	Initial Carbon Source (7 hr)	Shift Second Carbon Source	Germlings with >1 Nucleus (%)*^a^*
*ΔptpB*	Glu	No shift (0 hr)	7
	Glu	CA (3 hr)	54
	Glu	CA + HUb (3 hr)	6
	CA + HU^b^	No shift	8
	CA + HU^b^	CA	76
	CA + HU^b^	Glu	78
*ΔmsgA*	Glu	No shift (0 hr)	4
	Glu	CA (3 hr)	3
	Glu	CA (5 hr)	78
	Glu	CA + HUb (3 hr)	1
	Glu	CA + HUb (5 hr)	3
	CA + HU^b^	No shift (0 hr)	7
	CA + HU^b^	CA (3 hr)	78
	CA + HU^b^	Glu (3 hr)	77
*ΔppsA*	Glu	No shift (0 hr)	8
	Glu	CA (3 hr)	63
	Glu	CA + HUb (3 hr)	70
	CA + HU^b^	No shift (0 hr)	2
	CA + HU^b^	CA (3 hr)	83
	CA + HU^b^	Glu (3 hr)	84

a 100 germlings were counted for each experiment and the percentage having completing at least one nuclear division.

b Germlings were incubated in the presence of 25 mM hydroxyurea (HU).

As a control, the reciprocal experiment was performed in which conidia were primarily incubated in CA plus HU to synchronize the conidia in S phase and then shifted to glucose or CA without HU (Table 4). Releasing cell cycle in CA without HU resulted in 76–83% of germlings from the three mutants having multiple nuclei. Similarly, after shifting the three mutants to glucose, between 78% and 84% of germlings also underwent mitotic division. These finding suggest that the *ΔptpB* and *ΔmsgA* conidia possess a defect that cause them to be unable to exit from G1-S phase during germination on glucose. In addition, *ΔmsgA* also showed a delay in the reactivation of cell cycle. In contrast, the *ΔppsA* conidia, when incubated in glucose and transferred to CA plus HU, were able to increase nuclei number, suggesting that this mutant contained a defect after S phase, possibly mitosis, which inhibited germination on glucose. Collectively, these results demonstrate that the seven NPP mutants possessed defects involved in the detection of glucose and in the initiation of events essential for germination. Subsequently, the influence of the seven NPPs on the second phase of germination and outward growth on glucose was assessed.

### NPPs influence trehalose levels in conidia and its breakdown on germination

**Figure 3:**
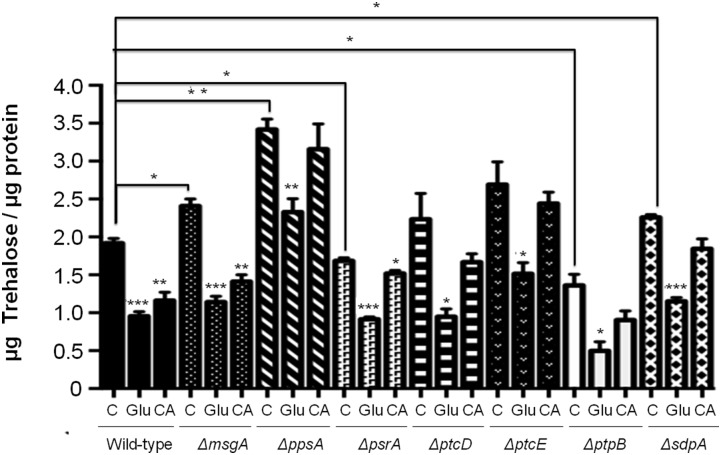
Conidial trehalose levels during germination on glucose and CA. The 5×10^7^ conidia from seven NPP deletion mutants and wild-type strain were germinated on minimal media plus glucose or CA, as a sole carbon source, for 150 min at 37°. The total protein lysates from conidia were used to measure the trehalose and were compared to standard curve. C = control (fresh conidia in water); Glu = glucose; CA = hydrolyzed casein. Presented is the average (± standard deviation) of three independent experiments. Significance = *P < 0.05, ** *P* < 0.01, and *** *P* < 0.001. The lines above the bars show the comparisons between the wild-type and the mutant controls or between the controls and Glu/CA treatments for each specific strain.

Trehalose is stored within conidia and is metabolized during the isotropic growth phase at the onset of germination trehalose (D’Enfert 1997; D’Enfert *et al.* 1999; Fillinger *et al.* 2002) prior to the uptake and metabolism of the external carbon source, which triggered the breaking of dormancy (MacCabe 2003). Subsequently, trehalose levels within dormant conidia and after germination in glucose or CA were investigated (Figure 3). The Δ*ppsA* and Δ*ptcE* mutants showed higher trehalose levels under all conditions. Trehalose levels in the wild-type strain declined after germination in either glucose or CA. All seven NPP mutants also showed a decline in trehalose level during germination on glucose. However, excluding, Δ*msgA*, the remaining six NPP mutants showed no significant decline in trehalose level during germination in CA. Therefore, it would appear that these six NPP mutants mobilized conidial trehalose stores to fuel germination on glucose, but downstream defects in saccharide metabolism impeded successful germination. Alternatively, during germination on CA, six NPP mutants did not mobilize conidial trehalose stores and possibly redirected metabolism toward the utilization of CA and successful germination.

### NPPs influence glucose and oxygen consumption

**Figure 4:**
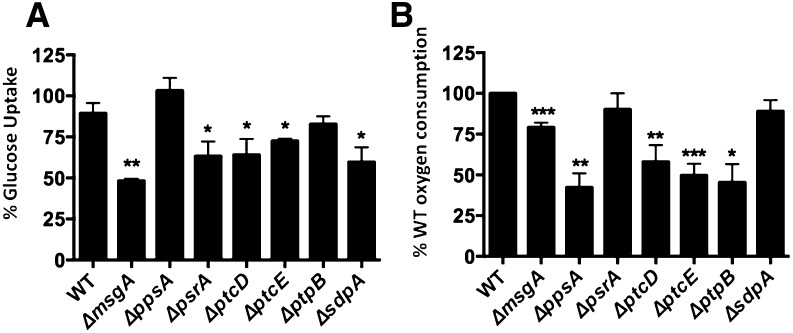
Glucose and oxygen consumption in the seven NPP mutants and the wild-type strain. Conidia were inoculated in minimal medium supplemented with CA for 24 hr, before mycelia were transferred to minimal medium plus glucose for 24 hr. (A) Glucose concentrations of the supernatant were measured and calculated using the difference between the glucose concentration at time point 0 hr (100% glucose) and after 24 hr. (B) The rate of oxygen consumption [ng atoms O/min/dry weight (mg)]. Presented are the percentages of glucose and oxygen consumption in the NPP mutants. Results are the average of three independent experiments (± square deviation). Significance = *P < 0.05, **P < 0.01, and ***P < 0.001.

A defect in the uptake and/or metabolism of saccharides may have contributed to the inability to germinate on such carbon sources. Subsequently, the capacity of the seven NPP mutants to consume glucose and oxygen was determined (Figure 4). The NPP mutants and the wild-type strain were pre-grown in media containing CA and then transferred to media containing glucose as a sole carbon source. After 24 hr, the amount of residual glucose in the media was measured. The wild-type strain plus the *ΔptpB* and *ΔppsA* mutants consumed all the available glucose, whereas the remaining five NPP mutants (*ΔmsgA*, *ΔptcD*, *ΔptcE*, *ΔpsrA*, and *ΔsdpA*) showed an approximate 60% reduction in glucose consumption (Figure 4A).

**Figure 5:**
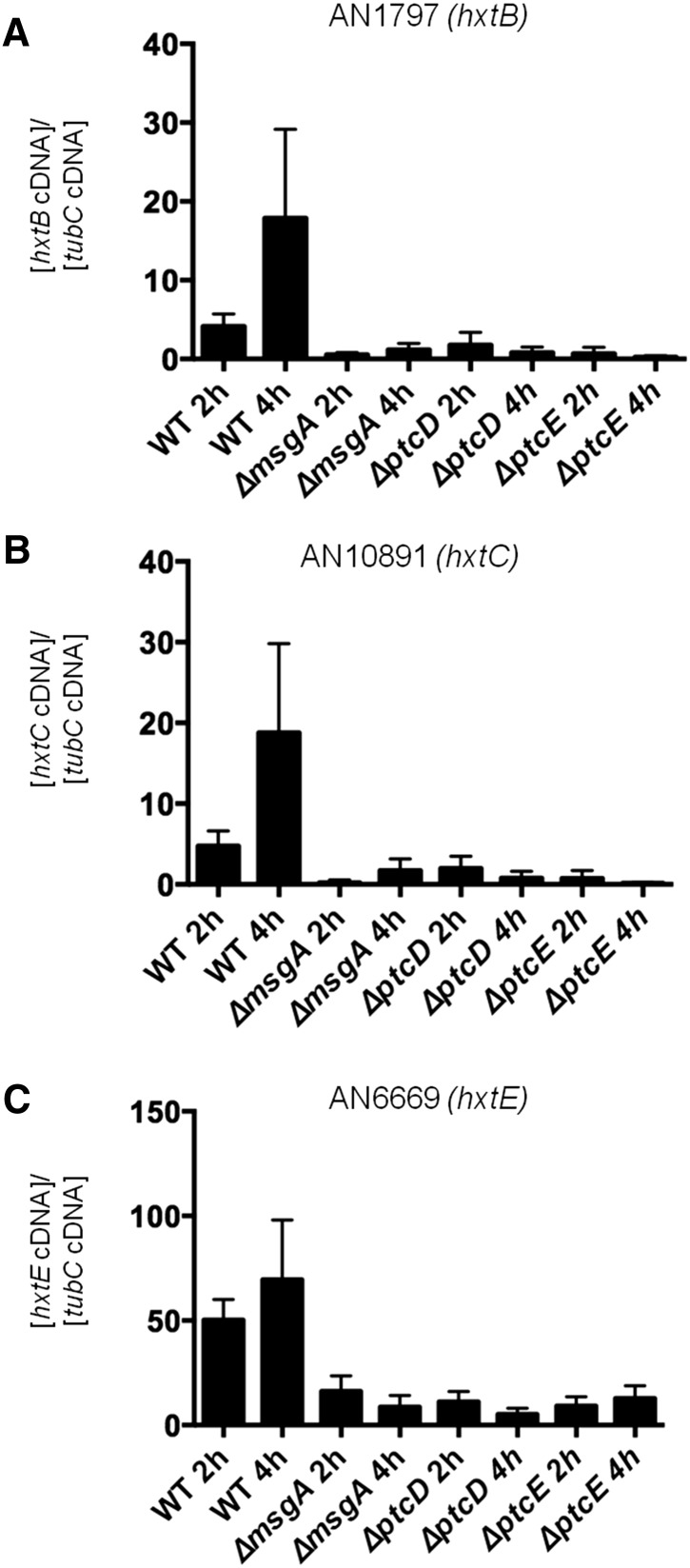
The expression of three major glucose transporter encoding genes in three NPP mutants and the wild-type strain (FGSG A4). Mycelia were grown in minimal medium plus CA for 24 hr before being transferred to minimal medium plus glucose for 2 or 4 hr. Gene expression of (A) AN1797 (*hxtB*), (B) AN10891 (*hxtC*), and (C) AN6669 (*hxtE*) was measured by RT-qPCR and values were normalized to the expression of tubC. The values are the average of three independent experiments (± standard deviation). Glucose transporter encoding gene expression was significantly reduced in all the NPP mutants (*P* < 0.05).

Simultaneously, the consumption of oxygen by the seven NPP mutants was determined. The *ΔpsrA* and *ΔsdpA* mutants consumed oxygen at a similar rate as the wild-type strain, whereas the *ΔmsgA*, *ΔppsA*, *ΔptcD*, *ΔptcE*,and *ΔptpB* showed a significant reduction in oxygen consumption (Figure 4B). Collectively, these data show that the NPP mutants that were more resistant to 2-DG (*ΔmsgA*, *ΔptcB*, and *ΔptcE*) had decreased consumption and/or uptake of glucose, coupled with a reduction in oxygen consumption, which conveyed 2-DG resistance. In accordance, a reduction in the expression of three glucose transporters, *hxtB* (AN1797), *hxtC* (AN10891), and *hxtE* (AN6669), was observed in the *ΔmsgA*, *ΔptcB*, and *ΔptcE* strains after transfer from CA to glucose-rich media for 2 hr and 4 hr, suggesting that glucose uptake and signaling was impaired (Figure 5, A–C). Therefore, all seven NPP mutants were impaired in glucose uptake and/or respiration, suggesting that these NPPs perform additional roles in regulating metabolism beyond germination.

### Intracellular ethanol, glycerol, trehalose, and pyruvate levels imply shifts in primary metabolism in the NPP mutants

**Figure 6:**
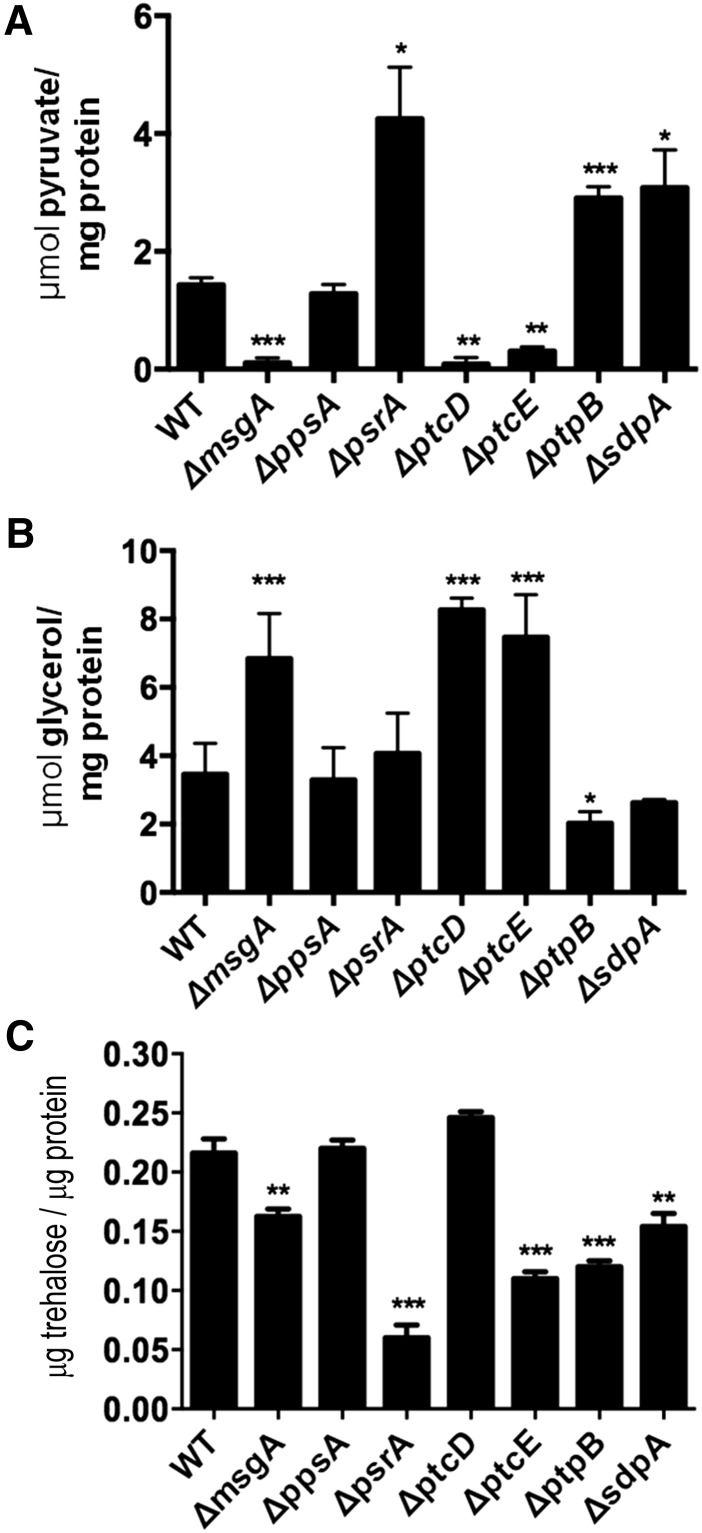
Intracellular pyruvate (A), glycerol (B), and trehalose (C) levels in the mycelia of the NPP mutants and wild-type strain during growth on glucose as a sole carbon source. Conidia were incubated in minimal medium plus CA for 24 hr. Subsequently, the mycelia were transferred to minimal medium plus 1% glucose for 24 hr. The average concentrations were determined from three independent experiments (± standard deviation). The concentrations of glycerol, trehalose, and pyruvate are presented in μmol.mg^−1^. Significance = *P < 0.05, **P < 0.01, and ***P < 0.001.

The NPP mutants were unable to grow directly on glucose as a sole carbon source. However, when transferred from CA to glucose containing media, all strains were able to consume glucose and respire to an extent. Therefore, to assess where glucose metabolism had been redirected, the intracellular glycerol, trehalose, and pyruvate content were measured. The NPP mutants and the wild-type strain were grown on CA and then transferred to media containing glucose as a sole carbon source. Intracellular pyruvate levels in the *ΔmsgA*, *ΔptcD*, and *ΔptcE* mutants had significantly decreased whereas in the *ΔpsrA*, *ΔptpB*, and *ΔsdpA* mutants pyruvate levels had increased when compared with the wild-type strain (Figure 6A), No change was observed in the *ΔppsA* mutant (Figure 6A). Intracellular trehalose was reduced in the *ΔmsgA*, *ΔptcE*, *ΔpsrA*, *ΔptpB*, and *ΔsdpA* mutants, whereas *ΔppsA* and *ΔptcD* were similar to the wild-type strain (Figure 6B). The three NPP mutants with reduced pyruvate levels, *ΔmsgA*, *ΔptcD*, and *ΔptcE*, showed an approximate two-fold increase in intracellular glycerol levels compared with the wild-type strain (Figure 6C). These results suggest that glucose metabolism is directed toward glycerol production in the *ΔmsgA*, *ΔptcD*, and *ΔptcE* mutants, which seem unable to produce significant amounts of pyruvate. In contrast, the *ΔptpB* mutant had reduced glycerol levels, suggesting that glucose metabolism was redirected toward fermentation. Hence, ethanol production in the *ΔptpB* mutant was measured, revealing a three-fold increase in ethanol production (0.09 and 0.03 g.L^−1^ ethanol in the *ΔptpB* and wild-type strains, respectively. This result suggests that glucose metabolism is directed toward fermentation in the *ΔptpB* mutant.

### Mapping the block in glucose metabolism reveals TCA cycle defects in the NPP mutants

The observation that the seven NPP mutants were unable to germinate on saccharide or alcohol carbon sources (*e.g.*, glucose, xylose, glycerol, acetate, and ethanol; Figure 2) but could germinate and grow on CA or tributyrin suggested that these strains had defects in glycolysis, gluconeogenesis, or the TCA cycle. Hydrolyzed casein contains a mixture of amino acids that can be used by *A. nidulans* for carbon metabolism and growth. Individual amino acids enter the TCA cycle at one or two specific points from which six TCA precursors can be formed: pyruvate (from alanine, glycine, threonine, cysteine, serine, and tryptophan), acetyl-CoA (from isoleucine, leucine, phenylalanine, tyrosine, and tryptophan), alpha-ketoglutarate (from arginine, proline, histidine, and glutamine), succinyl-CoA (from isoleucine, valine, methionine, and threonine), fumarate (from tyrosine and phenylalanine), and oxalacetate (from asparagine and aspartate) (Nelson *et al.* 2008). The triglyceride called tributyrin is an ester of glycerol and butyric acid that can be degraded by β-oxidation into the TCA intermediate acetyl-CoA, citrate, and succinyl-CoA. Interestingly, however, the seven NPP mutants were unable to grow on glycerol, whereas butyric acid alone was toxic (data not shown) to all NPP mutants and the wild-type strain at multiple concentrations (0.25%, 0.50%, and 1%), as previously described by Pohl *et al.* (2011).

**Figure 7:**
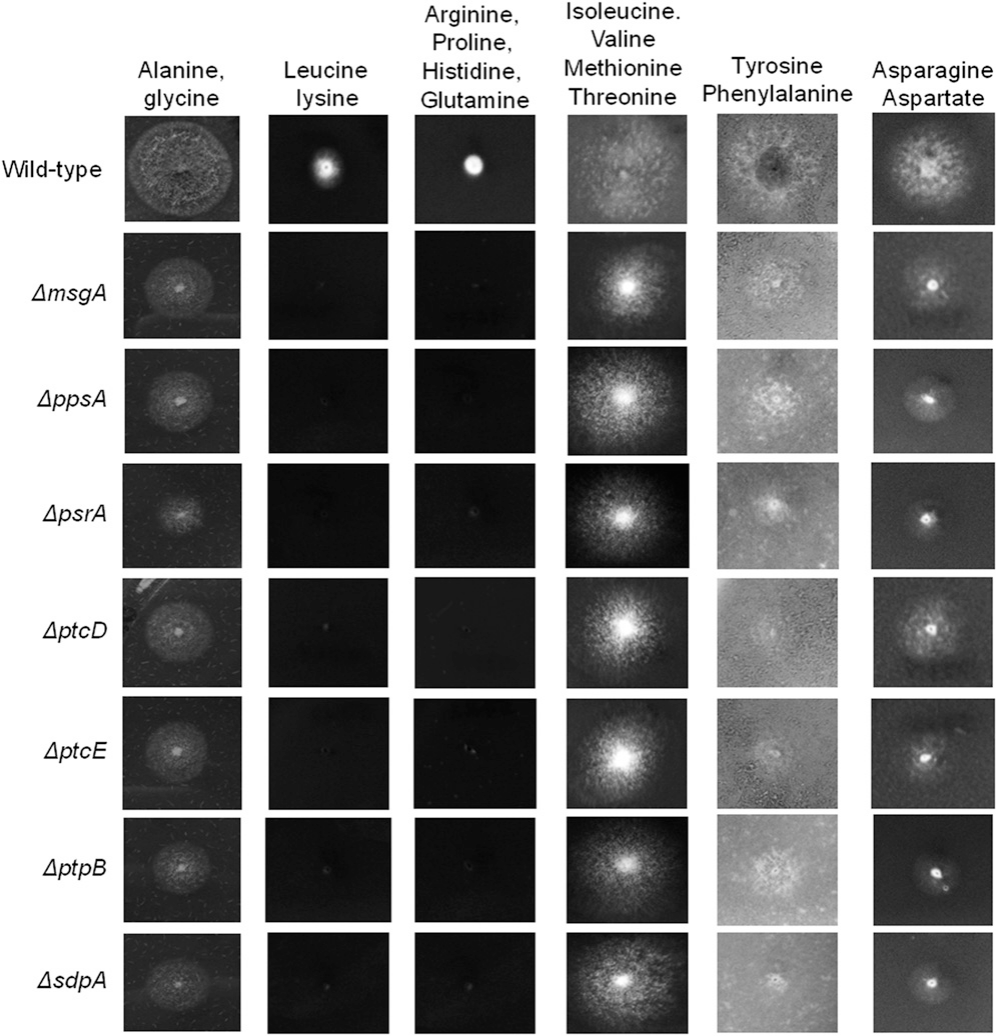
Growth of the seven NPP null mutants and the wild-type strain (FGSC A4) on a combination of amino acids (50 mM each amino acid) that enter the TCA cycle at specific points: pyruvate (alanine and glycine), acetyl-CoA (leucine and lysine), ketoglutarate (arginine, proline, histidine and glutamine), succinyl-CoA (isoleucine, valine, methionine and threonine), fumarate (tyrosine and phenylalanine), and oxaloacetate (asparagine and aspartate). All strains were grown at 37° for 96 hr.

Consequently, groups of amino acids that could only be metabolized via a specific route into glycolysis, gluconeogenesis, and the TCA cycle were utilized to map the metabolic block in carbon metabolism (Figure 7). All the NPP mutants were able to utilize groups of amino acids that acted as precursors for pyruvate, but the NPP mutants were unable to utilize amino acid precursors for acetyl-CoA or α-ketoglutarate, whereas growth was restored on amino acid precursors for succinyl-CoA, fumarate, and oxalacetate (Figure 7). This suggested that the block in glucose metabolism was in the oxidative decarboxylation of α-ketoglutarate to succinyl-CoA, which is catalyzed by the α-ketoglutarate dehydrogenase (α-KGDH).

**Figure 8:**
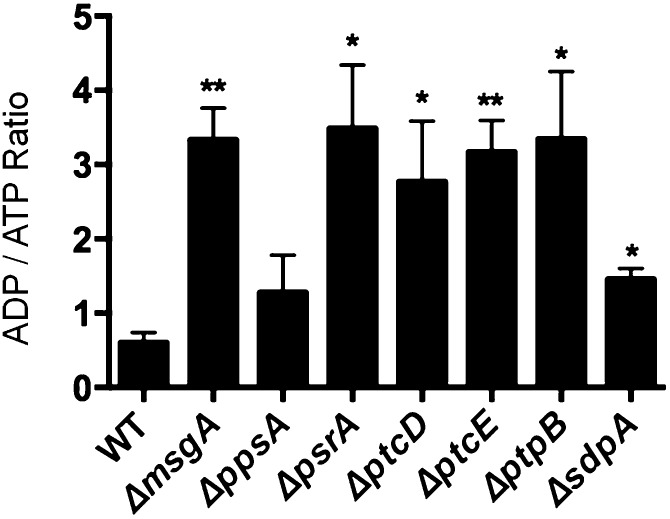
ADP/ATP ratio. The seven NPP deletion mutants were grown on minimal media plus CA for 24 hr and transferred to minimal media plus glucose for another 24 hr. The total protein lysates (10 μg) from mycelia were used to quantify the ADP/ATP ratio using the ADP/ATP ratio assay kit (Sigma). Significance = *P < 0.05, **P < 0.01, and ***P < 0.001.

The NPP mutants were shown to have defects the TCA cycle, inhibiting oxidative phosphorylation, which would therefore impede the production of ATP. Subsequently, the ADP/ATP ratio was measured under the same conditions as the glycerol, trehalose, and pyruvate measurements (Figure 8). The *ΔppsA* mutant had a similar ADP/ATP ratio to the wild-type strain, whereas the *ΔmsgA*, *ΔpsrA*, *ΔptcD*, *ΔptcE*, *ΔptpB*, and *ΔsdpA* mutants all had an increased ADP/ATP ratio. This showed that these six NPP mutants had reduced ATP level representative of a lower energetic state due to their defects in the TCA cycle.

Groups of amino acids were used to minimize the possible impact of defects in amino acid catabolism. In addition, the growth and germination of all the NPP deficient strains on minimal media plus CA were comparable to the wild-type, suggesting that there was no major defect in amino acid metabolism, whereas all NPP deficient strains also showed reduced growth rates on glucose (after germination). This strongly suggested that the metabolic defect(s) truly resided in carbon metabolism.

Collectively, these results demonstrate that multiple phosphatases influence primary carbon metabolism, whereas the deletion of these NPP-encoding genes impedes the production of energy and carbon compounds required for growth. In addition, these data implicated a crucial role for the α-KGDH in the regulation of conidial germination.

### NPPs influence alpha-ketoglutarate dehydrogenase activity

**Figure 9:**
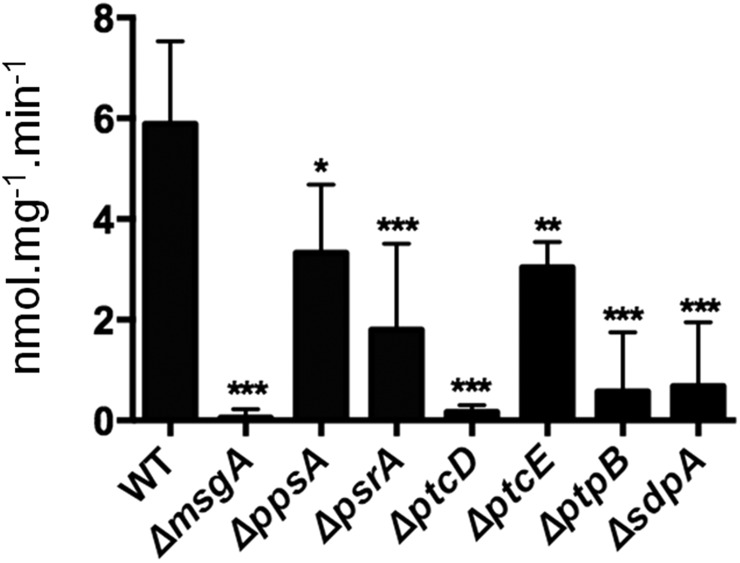
Alpha-ketoglutarate dehydrogenase activity in the seven NPP null mutants and the wild-type strain (TN02A3). Conidia were germinated in minimal medium plus CA for 24 hr and then transferred to minimal medium plus glucose for an additional 24 hr. The presented average alpha-ketoglutarate dehydrogenase activity represents three independent experiments (± square deviation). Significance = *P < 0.05, **P < 0.01, and ***P < 0.001.

In *S. cerevisiae*, two α-KGDH isoforms are encoded by the *KGD1* and *KGD2* genes (Repetto and Tzagoloff 1989, 1990). In *A. nidulans*, the genes *kgd*A (AN5571) and *kgdB* (AN3466) were identified with high identity to the two *S. cerevisiae* genes *KGD1* (e-value, 0.0; 65% identity; 79% similarity) and *KGD2* (e-value, 3.e-133; 60.2% identity; 74.1% similarity). To further support the hypothesis that the seven NPP mutants possessed defects in the ability to convert α-ketoglutarate to succinyl-CoA, α-KGDH activity was measured. The NPP mutants and the wild-type strain were grown in CA and then transferred to glucose-rich media for an additional 24 hr. All seven of the NPP mutants had decreased α-KGDH activity when compared to the wild-type strain (Figure 9). This implied that NPP mutants were impaired in the conversion of α-ketoglutarate to succinyl-CoA and unable to grow on carbon sources prior to succinyl-CoA, due to a de-regulation of α-KGDH activity.

### Microarray analyses revealed that PtpB influences the transcriptional response to growth on glucose and alteration to primary metabolism

In this study, the *ΔptpB* strain was shown to be unable to grow on saccharides including glucose, to have a defect in cell cycle progression, and to have extreme sensitivity to 2-DG during germination, whereas mycelia were shown to have reduced respiration and increased resistance to 2-DG. To ensure that the ∆*ptpB* strain (containing ∆*ptpB*::*pyrG^Af^* deletion cassette) did not possess additional mutations that impacted on carbon metabolism, it was sexually crossed with a strain containing the nonfunctional *pyrG89* mutation, which is auxotrophic for uridine and uracil. The subsequent ∆*ptpB*::*pyrG^Af^* progeny, which were uridine and uracil prototrophs, were selected. Phenotypic investigation of the progeny revealed ∆*ptpB*::*pyrG^Af^* cassette co-segregated with an inability to germinate and grow on glucose (data not shown), strongly indicating that PtpB is responsible for the observed phenotypes.

**Figure 10:**
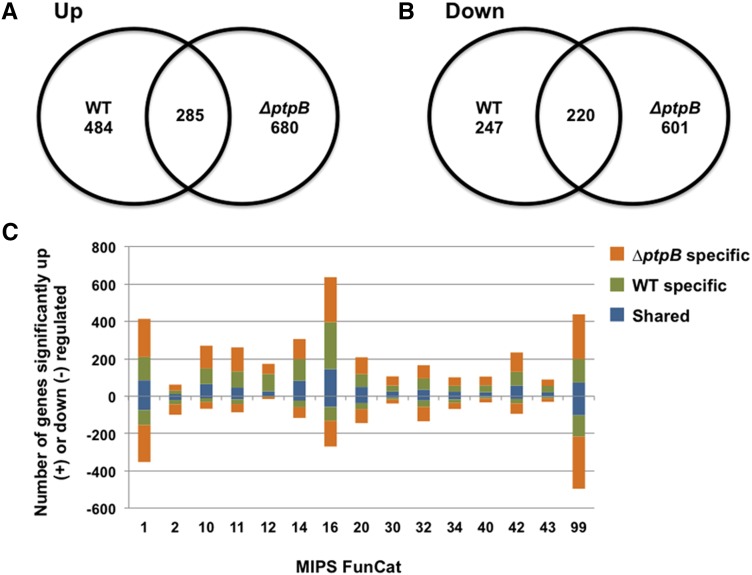
Genome-wide transcriptional profiling of the *ΔptpB* and wild-type (TN02A3) strains after transfer from CA to glucose as a sole carbon source for 4 hr. (A and B) Venn analysis of the genes differentially upregulated and downregulated after transfer to glucose (*P* < 0.01) identified the strain-specific transcriptional modulations. (C) MIPS Functional categorization reveals the functional profile of the strain-specific transcriptional modulations.

As an initial step to characterize the effect of the phosphatase mutants in regulating glucose metabolism, we investigated the role of PtpB by using genome-wide microarray analyses. The main objective was to identify strain-specific transcriptional differences during the metabolism of CA or glucose as the sole carbon source. The wild-type and *ΔptpB* strains were grown for 24 hr on CA and then transferred to glucose media for 4 hr. Genes that were differentially expressed between carbon sources in an individual strain were identified (*P* < 0.01) and subsequently divided into genes that were upregulated or downregulated after transfer to glucose. Strain-specific differences in the modulation of gene expression were identified via Venn analyses (Figure 10, A and B, Table S2, Table S3, Table S4, Table S5). An overview of the functional profile of the strain-specific gene sets was revealed via observing alterations in FunCat representation and the identification of over-represented gene ontologies (GO terms; *P <* 0.05) (Figure 10C, Table 5).

**Table 5:** Summary of the strain-specific over-represented gene ontologies

GO Term	Description	*P**^a^*	FDR*^b^*	Class*^c^*
WT-specific, upregulated				
GO:0006407	rRNA export from nucleus	0.000135	0.010559	BP
GO:0000462	Maturation of SSU-rRNA from tricistronic rRNA transcript (SSU-rRNA, 5.8S rRNA, LSU-rRNA)	5.61E-05	0.006168	BP
GO:0006413	Translational initiation	3.6E-06	0.000648	BP
GO:0000028	Ribosomal small subunit assembly	0.000276	0.015596	BP
GO:0006448	Regulation of translational elongation	0.000886	0.038994	BP
GO:0006999	Nuclear pore organization	7.22E-05	0.006502	BP
GO:0005663	DNA replication factor C complex	0.004221	0.037211	CC
GO:0005789	Endoplasmic reticulum membrane	0.000398	0.004731	CC
GO:0005852	Eukaryotic translation initiation factor 3 complex	0.001067	0.01154	CC
GO:0031389	Rad17 RFC-like complex	0.001792	0.018153	CC
GO:0022627	Cytosolic small ribosomal subunit	3.43E-12	3.26E-10	CC
GO:0022625	Cytosolic large ribosomal subunit	1.72E-09	8.21E-08	CC
GO:0005643	Nuclear pore	0.000129	0.002363	CC
GO:0016272	Prefoldin complex	0.004221	0.037211	CC
GO:0071212	Subsynaptic reticulum	0.000353	0.004314	CC
GO:0030686	90S preribosome	0.00044	0.005109	CC
GO:0031391	Elg1 RFC-like complex	0.001792	0.018153	CC
GO:0003735	Structural constituent of ribosome	1.39E-11	1.02E-08	MF
GO:0003743	Translation initiation factor activity	0.000164	0.015023	MF
WT-specific, downregulated			
None				
*ΔptpB*-specific, upregulated			
GO:0034645	Cellular macromolecule biosynthetic process	9.45E-05	0.01169	BP
GO:0000466	Maturation of 5.8S rRNA from tricistronic rRNA transcript (SSU-rRNA, 5.8S rRNA, LSU-rRNA)	1.89E-05	0.003119	BP
GO:0000469	Cleavage involved in rRNA processing	0.000211	0.022581	BP
GO:0042273	Ribosomal large subunit biogenesis	0.000248	0.024558	BP
GO:0044452	Nucleolar part	8.91E-06	0.000386	CC
GO:0030687	Preribosome, large subunit precursor	4.7E-06	0.000227	CC
GO:0032040	Small-subunit processome	0.000722	0.021471	CC
GO:0030686	90S preribosome	0.000168	0.005328	CC
*ΔptpB*-specific, downregulated			
GO:0006575	Cellular modified amino acid metabolic process	0.000738	0.042632	BP
GO:0044271	Cellular nitrogen compound biosynthetic process	0.000998	0.04817	BP
GO:0006744	Ubiquinone biosynthetic process	0.00084	0.042632	BP
GO:0033539	Fatty acid beta-oxidation using acyl-CoA dehydrogenase	0.000597	0.042044	BP
GO:0009083	Branched chain family amino acid catabolic process	5.89E-07	0.000104	BP
GO:0017086	3-methyl-2-oxobutanoate dehydrogenase (lipoamide) complex	0.000155	0.018489	CC

Summary of the strain-specific over-represented gene ontologies (GO terms) in the genes differentially modulated transcriptionally after transfer from CA to glucose as a sole carbon source. The complete list of strain-specific and nonspecific GO terms is available in Table S2, Table S3, Table S4, and Table S5.

a *P* for the Fishers exact test.

b False discovery rate.

c GO term classifications: BP, biological process; CC, cellular component; MF, molecular function.

The two strains exhibited distinct transcriptional profiles as represented by the small proportion of genes that were modulated in both strains after transfer to glucose. Overall, the Δ*ptpB* mutant modulated the expression of a higher number of genes than the wild-type strain, particularly those involved in metabolism. Both strains showed an up-egulation of genes involved in the modulation of cell cycle and replication after transfer to glucose. The wild-type strain specifically showed an upregulation of processes related to protein translation, whereas the Δ*ptpB* strain upregulated different genes involved in ribosome biogenesis and downregulated alternative carbon usage when grown on glucose.

**Figure 11:**
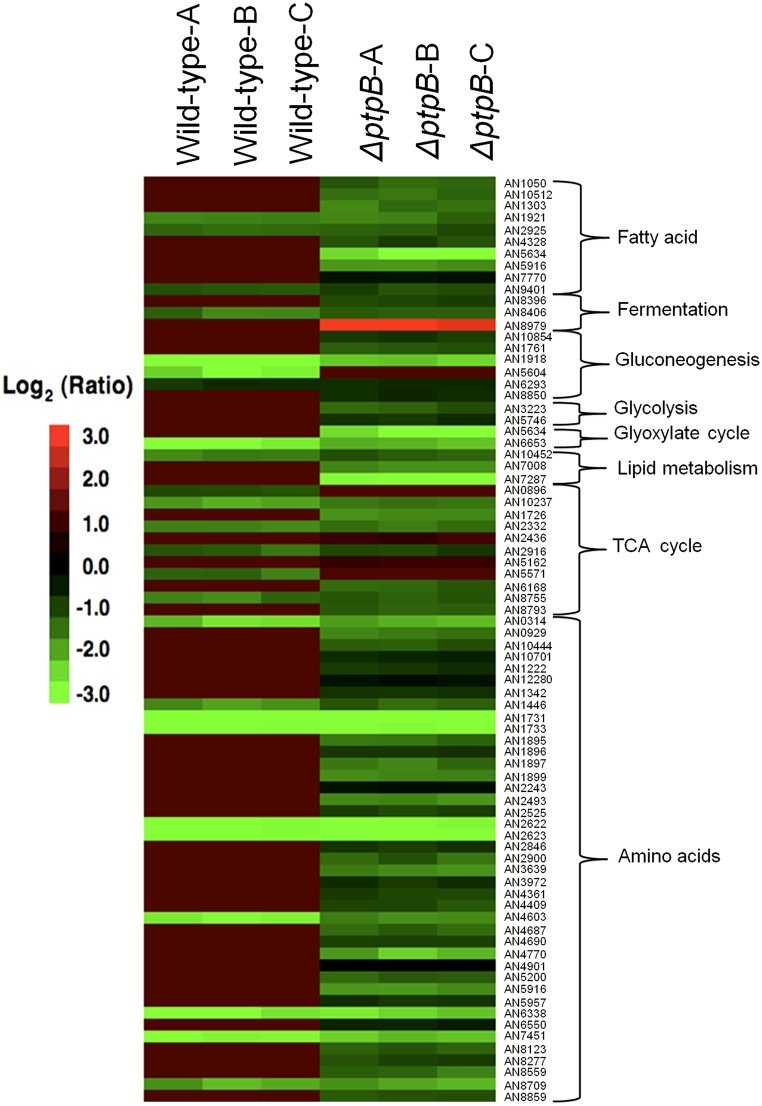
Heatmap displaying the transcriptional profiles of selected genes encoding for proteins involved in glycolysis, gluconeogenesis, fermentation, and the TCA cycle in the three independent replicates of the wild-type and *ΔptpB* strains.

Subsequently, the transcriptional modulation of genes specifically involved in primary carbon metabolism, including glycolysis, gluconeogenesis, TCA cycle, glycoxylate cycle, alcohol fermentation, amino acid catabolism, lipid metabolism, and fatty acid beta-oxidation, were inspected (Figure 11). Several key steps in carbon metabolism were de-regulated in the Δ*ptpB* mutant when compared to the wild-type strain. These included the following: the *pfkA* phosphofructokinase, involved in glycolysis; the *acuG* fructose-1,6-bisphosphatase, which is a key regulatory enzyme in gluconeogenesis; the *acuK* transcription factor that regulates gluconeogenesis; the *FbaA* fructose-bisphosphate aldolase, which is important for growth on nonsugar carbon sources; subunits of the pyruvate dehydrogenase complex (3-methyl-2-oxobutanoate dehydrogenase, alpha-keto acid dehydrogenase E1 and E2; AN1726, AN8559, AN3639) involved in amino acid catabolism; and the *hadA* hydroxy-acyl-CoA dehydrogenase, the *echA* enoyl-CoA hydratase, and *mthA* ketoacyl-CoA thiolase, required for fatty acid beta-oxidation and amino acid catabolism. In addition, specifically in the *ΔptpB* strain, an upregulation of the alcohol regulator *alcR* and the alcohol dehydrogenase gene *alcA* was observed.

**Figure 12:**
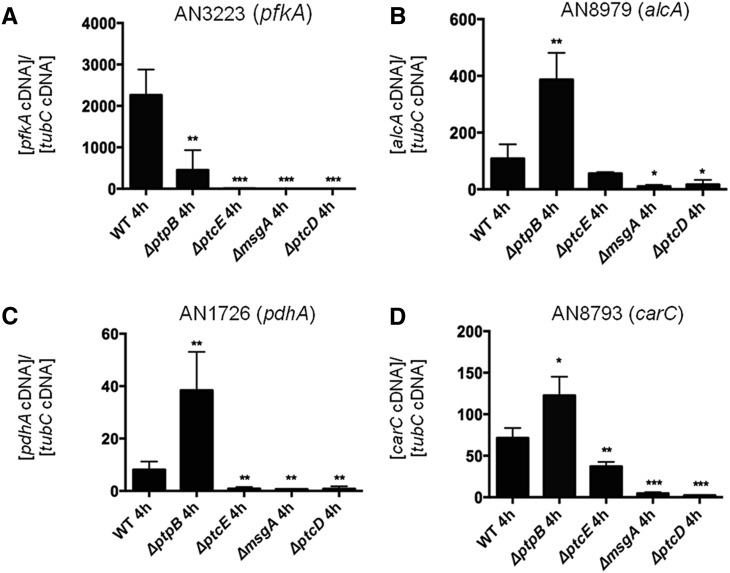
RT-qPCR analyses of genes encoding for metabolic enzymes involved in primary carbon metabolism in the wild-type, *ΔptpB*, *ΔmsgA*, *ΔptcD*, and *ΔptcE* strains after transfer from CA to glucose as a sole carbon source. (A) AN3223 (*pfkA*), (B) AN8979 (*alcA*), (C) AN1726 (*pdhA*), and (D) AN8793 (*carC*).

Subsequent RT-qPCR analyses of genes encoding for metabolic enzymes involved in primary carbon metabolism (*pfkA*, *alcA*, *pdhA*, and *carC* encoding 6-phosphofructokinase, alcohol dehydrogenase, alpha subunit of pyruvate dehydrogenase, and succinate dehydrogenase, respectively), which were differentially regulated in the *ΔptpB* strain, were performed for the wild-type, *ΔptpB*, *ΔmsgA*, *ΔptcD*, and *ΔptcE* strains. These analyses confirmed the results observed in the microarray and demonstrated that primary carbon metabolism was similarly transcriptionally altered in the *ΔmsgA*, *ΔptcD*, and *ΔptcE* strains (Figure 12, A–D). These results reveal how PtpB influences the transcriptional regulation of multiple key steps in primary carbon metabolism.

## Discussion

Conidial germination is an extremely complex biological process that requires the breaking of dormancy, morphogenesis, the reactivation of the cell cycle, the biosynthesis of new cell components, adaptations to stress, and the shifting of metabolism toward energy, yielding reactions. In *A. nidulans*, conidia can germinate in the presence of water, salts, and a carbon source while being able to utilize a diverse array of simple and complex carbon sources. Hence, carbon sensing has a profound impact on *A. nidulans* germination, growth, and the aforementioned biological processes (Fillinger *et al.* 2002; Lafon *et al.* 2005, 2006). However, the signaling mechanisms that coordinate these processes are unclear. In the present study, seven phosphatases were identified to perform fundamental roles during conidial germination on saccharide and alcohol carbon sources, broadly influencing morphological adaptations, metabolism, and cell cycle (Table 6). This evidence represents the basis for the future dissection of carbon sensing and the signaling pathways involved in germination.

**Table 6:** Summary of the phenotypic characterization of the *A. nidulans* phosphatase mutants

	Fungal Strain							
Trait	WT	*ΔmsgA* AN4544	*ΔppsA* AN0129	*ΔpsrA* AN10077	*ΔptcD* AN0914	*ΔptcE* AN5722	*ΔptpB* AN4896	*ΔsdpA* AN10138
Direct growth on glucose (Fig. 2)	+	−	−	−	−	−	−	−
After germination growth on glucose (Fig. 2)	+	+	+	+	+	+	+	+
Pre-CA and growth on glucose (Table 1)	0.056	0.033**	0.032**	0.033**	0.033**	0.033**	0.030***	0.035***
Isotropic growth on glucose (%) (Table 3)	202	75****	105**	26****	24****	0****	105****	46****
Cell cycle defect (Table 4)	N/A	G1-S	M	N/A	N/A	N/A	G1-S	N/A
Trehalose mobilization on Glucose (Fig. 3)	+	+	+	+	+	+	+	+
Trehalose mobilization on CA (Fig. 3)	+	+	−	−	−	−	−	−
Before germination 2-DG (mM) (Fig. S1)	0.08	0.1 (++)	0.1 (+)	0.08	0.1 (++)	0.1 (++)	0.05 (−)	0.08 (−)
After germination 2-DG (mM) (Fig. S2)	0.01	0.04 (++)	0.04 (++)	0.025 (+)	0.04 (++)	0.04 (++)	0.04 (++)	0.05 (+++)
Glucose consumption (% WT) (Fig. 4)	89.4	48.3**	103	63.3*	64.1*	72.6*	82.9	59.8*
Glucose respiration (% WT) (Fig. 4)	100	79.2***	42.4**	90.3	58.1**	49.8***	45.5*	89.1
Trehalose (μg trehalose/μg protein) (Fig. 6)	0.21	0.16**	0.22	0.06***	0.24	0.11***	0.12***	0.15**
Glycerol content (Fig. 6) (μmol/mg protein)	3.5	6.9***	3.3	4.1	8.3***	7.5***	2.0*	2.6
Pyruvate content (Fig. 6) (μmol/mg protein)	1.4	0.1***	1.3	4.3*	0.1*	0.3**	2.9***	3.1*
Glycolytic saccharide precursors (Fig. 2)	+	−	−	−	−	−	−	−
Nonsaccharide carbon sources (acetate, ethanol, glycerol) (Fig. 2)	+	−	−	−	−	−	−	−
Lipid/casein amino acids (Fig. 2)	+	+	+	+	+	+	+	+
Precursors for pyruvate (Fig. 7)	++	+	+	+	+	+	+	+
Precursors for acetyl-CoA or α-KGDH (Fig. 7)	+	−	−	−	−	−	−	−
Precursors pre-succinyl CoA (Fig. 7)	+	+	+	+	+	+	+	+
ADP/ATP ratio (Fig. 8)	0.6	3.4**	1.28	3.5*	2.8*	3.2**	3.4*	1.5*
Alpha-keto glutarate activity (Fig. 9) (nmol/mg.min)	5.9	0.1***	3.3*	1.8***	0.2***	3.0**	0.6***	0.7***

Level of statistical significance: *P < 0.05, **P < 0.01, and ***P < 0.001. Level and direction of alteration in growth: +/−.

The immediate response of conidia to the detection of conditions suitable for growth is represented by isotropic growth or swelling, which occurs via the absorption of water, and the reactivation of cell cycle (Bergen and Morris 1983; D’Enfert 1997; Momany and Taylor 2000). After a suitable carbon source has been detected, metabolism is rapidly switched from the basal utilization of nonsaccharide carbon sources via gluconeogenesis and the glycoxylate cycle to the use of trehalose and mannitol via glycolysis, providing the energy and carbon compounds required for growth (D’Enfert 1997; Hayer *et al.* 2013, 2014). The absence of any swelling in the *ΔptcE* mutant suggests that it is unable to detect glucose. Interestingly, the *ΔptcD* and *ΔpsrA* mutants also showed substantially reduced swelling and may also possess defects in glucose sensing. However, conidial trehalose stores were mobilized during germination on glucose for all NPP mutants, as in the wild-type strain. Therefore, the reduced isotropic growth in response to glucose could also be the consequence of issues in trehalose metabolism, considering that all strains are unable to utilize any of the tested saccharides, a defect that was bypassed during germination on CA.

The homologs of the identified *A. nidulans* PtcD and PtcE phosphatases in *S. cerevisiae*, Ptc5p and Ptc6p, are mitochondrial phosphatases that regulate the pyruvate dehydrogenase complex (Gey *et al.* 2008). The pyruvate dehydrogenase complex (PDC) acts as a metabolic switch in mammalian cells that regulates the utilization of alternative carbon sources. The PDC generates NADPH and acetyl-CoA from the oxidative decarboxylation of pyruvate and facilitates uptake into the mitochondria. The phosphorylation state of the PDC controls the flux through this irreversible reaction, thus directing metabolism toward the consumption of glucose in respiration or the preservation of glucose for gluconeogenesis (Wu *et al.* 2001). Under carbon limitation, the pyruvate dehydrogenase kinase phosphorylates and inactivates the PDC, conserving glucose and promoting fatty acid utilization (Wu *et al.* 2001). Disruption of the *A. nidulans* pyruvate dehydrogenase kinase, *pkpA*, resulted in the loss of an ability to grow on recalcitrant cellulose, which represents an alternative carbon source that requires the utilization of fatty acids for the synthesis of cellulolytic enzymes (Brown *et al.* 2013). Conversely, the present study showed that the disruption of the opposing phosphatases, *ptcD* and *ptcE*, resulted in the loss of germination, but not growth, on glucose. Therefore, the PDC appears to perform a fundamental role in the regulation of carbon metabolism in *A. nidulans*, which during germination acts as a metabolic switch promoting the use of glycolytic carbon sources.

Dormant *A. nidulans* conidia reside in the G1 phase of the cell cycle. Prior to germ-tube emergence, at least one full cycle has been completed and the number of nuclei has increased (Bergen and Morris 1983; Momany and Taylor 2000). Here, several *A. nidulans* phosphatase mutants showed conidial swelling on glucose but did not establish polarity. Subsequently, these mutants were shown to be unable to complete the cell cycle. These *A. nidulans* phosphatases included the homologs of the *S. cerevisiae* phosphatases, Ptp1p, Pps1p, and Msg5p. These three *S. cerevisiae* phosphatases are involved in the regulation of pathways influencing cell cycle and filamentation. Overexpression of Pps1p resulted in synchronous growth arrest in S phase and an abolishment of DNA synthesis (Ernsting and Dixon 1997). A screen for suppressors, identified *RAS2* as a multicopy suppressor, but not protein kinase A or the adenylate cylase, suggesting that Pps1p influences a PKA-independent Ras2 pathway (Ernsting and Dixon 1997). Msg5p performs a role in the recovery from G1 arrest after exposure to the mating pheromone, suggesting that the kinase FUS3p, which must be dephosphorylated for recovery from G1 arrest, is a substrate for Msg5p (Doi *et al.* 1994). Ptp1p is a phosphotyrosine-specific protein phosphatase that dephosphorylates a broad range of substrates, including the Fpr3p nucleolar peptidyl-prolyl *cis*-trans isomerase that acts as a transcriptional repressor, whereas Ptp1p is proposed to be a negative regulator of filamentation (Wilson *et al.* 1995). In addition, Ptp1p can complement a mutation in an endogenous *Schizosaccharomyces pombe* phosphatase, Cdc25, which regulates a key cell cycle regulator, Cdc2 (Hannig *et al.* 1993), suggesting the Ptp1p also influences cell cycle. Here, the putative homologs in *A. nidulans* were shown to also possess defects in cell cycle progression during germination on glucose, with the Δ*ppsA* strain possessing a block post S phase, possibly mitosis, and the Δ*ptpB* strain possessing a block in the transition between the G1 and S phases. Interestingly, Δ*msgA* also possessed a block between the G1 and S phases in addition to showing a delay in the reactivation of cell cycle progression, fitting with a putative role in the positive regulation of recovery from cell cycle arrest in G1.

The second developmental transition during germination in *A. nidulans* is represented by the establishment of polarity and germ-tube emergence (D’Enfert 1997). Filamentous growth in fungi is predominantly regulated by two mitogen-activated protein kinase (MAPK) cascades, termed the filamentous growth and cell wall integrity pathway (Rispail *et al.* 2009). In *A. nidulans*, the phosphatases regulating these MAPK cascades are unknown. In *S. cerevisiae*, two phosphatase paralogs, Msg5p and Sdp1p, regulate the phosphorylation state of the terminal kinase of the respective pseudohyphal growth/pheromone response and the CWI pathways, Fus3p/ Kss1p and Stl2p (Palacios *et al.* 2011). Here, seven phosphatase mutants were unable to establish polarity and germ-tube emergence. Two of the identified *A. nidulans* phosphatases that could not germinate on saccharides were putative homologs of Msg5p and Sdp1p, suggesting these strains may have had defects in filamentous growth and CWI pathways, a hypothesis that is supported by the delayed germination of Δ*msgA* on CA. The transition from conidia to hypha also exposes a germling to increased stress. This could contribute to the necessity for PsrA during *A. nidulans* germination on glucose, because the putative *S. cerevisiae* homolog Psr1p is involved in the regulation of Msn2-mediated general stress response, which is activated by dephosphorylation (Kaida *et al.* 2002). The faster growth rate on glucose compared to CA may enhance the exposure to stress and increase the requirement for PsrA during *A. nidulans* germination on glucose.

The α-KGDH is a TCA cycle enzyme that catalyzes the conversion of α-ketoglutarate, coenzyme A, and NAD^+^ to succinyl-CoA, NADH, and CO_2_ and requires thiamine pyrophosphate as a cofactor, which is inhibited by its end products (Tretter and Adam-Vizi 2004). The α-KGDH is distinct from the other TCA cycle enzymes because it is a highly regulated enzyme that controls the metabolic flux and energetic output (Hansford 1980). A transcriptomic analysis of *A. niger* revealed that the α-KGDH is upregulated during germination (Novodvorska *et al.* 2013). The present study showed that the seven phosphatase mutants, which were all unable to germinate and grow on carbon sources prior to succinyl-CoA, also possessed reduced α-KGDH activity. The α-KGDH blockage in TCA cycle pushes down cell energy levels as the oxidative phosphorylation pathway requires NADH, produced by TCA cycle, to sustain ATP production. The ADP/ATP ratios were increased in all the NPP mutants but decreased in the wild-type strain during growth on glucose. This implicates the α-KGDH as a fundamental step in the regulation of conidial germination in *A. nidulans*.

In summary, the transition from dormant conidia into growing filamentous hyphae requires the activation of numerous processes. The present study showed the diversity of roles performed by multiple phosphatases in regulating cell cycle, development, and metabolism in response to glucose and alternative carbon sources. In addition, this study highlighted the importance of several signaling pathways regulating filamentous growth, the action of the PDC as a metabolic switch controlling carbon usage, and the identification of the key function performed by the α-KGDH during germination. The dispersal and germination of conidia has a profound impact on pathogenicity and the industrial application of *Aspergillus* species. The food and feed industry commonly use preservatives against molds such as propionate, which inhibits the PDC (Brock and Buckel 2004). Hence, these novel insights into the fundamental roles of numerous phosphatases in germination and carbon sensing will prompt the future dissection of the implicated pathways and mechanisms in the most highly conserved morphogenic program in fungi while providing new avenues of research into the identification of inhibitors for fungal germination.
